# Exogenous activation of cannabinoid-2 receptor modulates TLR4/MMP9 expression in a spinal cord ischemia reperfusion rat model

**DOI:** 10.1186/s12974-020-01784-7

**Published:** 2020-04-06

**Authors:** Na Jing, Bo Fang, Zhe Li, Ayong Tian

**Affiliations:** Department of Anesthesiology, First Affiliated Hospital, China Medical University, 155 North Nanjing Street, Shenyang, 110001 Liaoning People’s Republic of China

**Keywords:** Cannabinoid-2 receptor, Ischemia reperfusion, Blood-spinal cord barrier, TLR4, MMP9, Astrocyte

## Abstract

**Background:**

Cannabinoid-2 receptor (CB2R) plays an important role in the cascading inflammation following ischemic injury. The toll-like receptors 4 (TLR4)/matrix metalloproteinase 9 (MMP9) signal pathway is involved in blood-brain barrier dysfunction induced by ischemia stroke. The aim of this study is to investigate the roles of exogenous activation of CB2R on attenuating neurological deficit and blood-spinal cord barrier (BSCB) disruption during rat spinal cord ischemia reperfusion (I/R) injury, through modulation of the TLR4/MMP9 axis.

**Methods:**

Animals were intraperitoneally pretreated with TLR4 inhibitor TAK-242, CB2R agonist JWH-133 with or without CB2R antagonist AM630, or equivalent volume of vehicle 1 h before undergoing 14-min occlusion of descending aorta or sham operation. One, two, three, and 7 days after reperfusion, hindlimb locomotor function was evaluated with Basso, Beattie, and Bresnahan (BBB) Locomotor Scale, BSCB integrity was detected by measurement of Evans blue (EB) extravasation and spinal cord edema. The protein expression levels of CB2R, tight junction protein Zonula occluden-1 (ZO-1), TLR4, MMP9, MyD88, NF-κB p65, and NF-κB p-p65 were determined by western blot. The MMP9 activity was analyzed by gelatin zymography. Double immunofluorescence staining was used to identify the perivascular localization of CB2R, TLR4, MMP9, and reactive astrocytes, as well as the colocalization of CB2R, TLR4, and MMP9 with reactive astrocytes.

**Results:**

JWH-133 pretreatment attenuated hindlimb motor functional deficit and BSCB leakage, along with preventing downregulation of ZO-1 and upregulation of TLR4/MMP9, similar to the effects of TAK-242 preconditioning. JWH-133 or TAK-242 pretreatment reduced the perivascular expression of TLR4/MMP9 and reactive astrocytes following injury. JWH-133 pretreatment also downregulated MyD88/NF-κB level, MMP9 activity, and the astrocytic TLR4/MMP9 after I/R injury.

**Conclusions:**

Exogenous activation of CB2R by JWH-133 attenuated neurological deficit and BSCB disruption after spinal cord I/R injury via inhibition of TLR4/MMP9 expression.

## Introduction

Spinal cord ischemia reperfusion (I/R) injury is well known as the most devastating complication in clinical thoracoabdominal aneurysm repair [1]. One of the major pathological changes in I/R injury is the impairment of blood-spinal cord barrier (BSCB), which plays a critical role in maintaining homeostasis of the spinal cord. Similarly to the blood-brain barrier, BSCB consists of continuous capillary endothelial cells lining spinal microvessels, perivascular endfeet of astrocytes, pericytes, and tight junctions between adjacent endothelial cells and basement membrane [2]. Astrocytes surround or closely associate with spinal capillaries. Reactive astrocytes can release chemical factors including inflammatory mediators to regulate blood-brain barrier permeability via interaction with the endothelium [3]. The opening of tight junction pathways has generally been found to be responsible for the increase of endothelial permeability. Among the tight junction related proteins, Zonula occluden-1 (ZO-1) has been evaluated as a marker protein [4]. Reduced expression of ZO-1 is closely related to BSCB breakdown in I/R injury [5]. Disruption of BSCB function and structure following I/R injury leads to neurological deficit. Mounting evidence indicates that BSCB integrity can be a neuroprotection target for reducing spinal cord I/R injury [5–9].

The receptor-mediated endocannabinoid system plays an important role in neuroprotection. The system consists of two main receptors, cannabinoid-1 receptor (CB1R) and cannabinoid-2 receptor (CB2R), endogenously-produced cannabinoids, and corresponding synthesizing and degrading enzymes [10]. In the spinal cord, CB1R is expressed constitutively in neurons, and can be induced by reactive astrocytes; while CB2R is strongly expressed in response to cascading inflammation associated with ischemic episode, mainly in astrocytes and immune infiltrates [11]. Our previous work showed that CB1R and CB2R were associated with BSCB disruption during spinal cord I/R injury [12]. CB1R increases rapidly to peak within 2 h to 6 h after ischemia, and returns to baseline at 24 h postischemia. CB2R increases at 24 h after ischemia [13, 14], and the hyperexpression can last for 28 days [11]. Considering that the most serious BSCB disruption occurred at 48 h after spinal cord I/R injury [15], we only aimed at CB2R in the current study. CB2R activation attenuates the cascading inflammation in hypoxia-ischemia events by downregulating reactive glial cells and reducing the release of inflammatory cytokines [16–18]. Previous studies have demonstrated CB2R activation contributes to ameliorating blood-brain barrier damage during cerebral I/R injury in vitro and in vivo [19–21]. CB2R agonist could modulate the BSCB permeability and the expression of tight junction proteins [22]. However, the mechanisms involved in the protective function of CB2R activation in BSCB breakdown following spinal cord I/R injury are still largely unknown.

Various studies have reported that toll-like receptors 4 (TLR4) are strongly associated with the inflammatory responses after I/R injury and mediate the motor dysfunction and BSCB disruption [7, 15, 23]. It has also been reported that brain ischemia induces TLR4 expressions in astrocytes, and that TLR4 activation leads to an astroglial polarization towards an inflammatory phenotype [24]. Astrocytes contribute to blood-brain barrier damage through activation of endothelial matrix metalloproteinase 9 (MMP9) [25]. MMP9 also influences BSCB integrity by degrading tight junction proteins, such as ZO-1, and modulate inflammation following tissue injury by facilitating immune cell infiltration [5, 8, 26, 27]. Interestingly, recent findings reveal that the TLR4/MMP9 signal pathway could regulate tight junctions in blood-brain barrier dysfunction induced by ischemic stroke [28]. Since exogenous activation of CB2R could inhibit TLR4 expression and hence have a significant effect on inflammatory responses in spinal cord injury [16], we investigated the hypothesis that exogenous activation of CB2R may have the potential protective effects against neurological deficit and BSCB disruption during spinal cord I/R injury through modulation of the TLR4/MMP9 axis.

In order to test our hypothesis, first, we studied the role of TLR4/MMP9 signal pathway by blocking the pathway with TLR4 inhibitor TAK-242, which could prevent TLR4 from interacting with its downstream targets [29]. Next, we used CB2R agonist JWH-133 preconditioning with or without the CB2R antagonist AM630. JWH-133 is a potent CB2R agonist (Ki = 3.4 nM) displaying approximately 200-fold selectivity over CB1 receptor [30, 31]. AM630 is a CB2R antagonist (Ki = 31.2 nM), which displays 165-fold selectivity over CB1 receptor [32, 33].

## Materials and methods

### Experimental animals

Male Sprague-Dawley rats, weighting from 200 to 230 g, were purchased from the animal center of the China Medical University. The experimental procedures were approved by the Ethics Committee for Animal Experimentation of China Medical University and in accordance with the Guide for the Care and Use of Laboratory Animals (National Institutes of Health, Bethesda, MD).

### Experimental protocol

Animals were randomly divided into one of five groups: Sham, I/R, I/R + TAK-242 (TAK), I/R+ JWH-133 (JWH), and I/R + JWH-133 + AM630 (JWH + AM) group, *n* = 45 for each group. As described in previous studies, 1 h prior to sham surgery or ischemia, intraperitoneal administrations were performed with TAK-242 (3 mg/kg) for the TAK group [34–36], JWH-133 (1 mg/kg) for the JWH group, JWH-133 (1 mg/kg), and AM630 (1 mg/kg) independently for the JWH + AM group [21], or equivalent volume of vehicle (DMSO) for the sham and I/R groups. The descending aorta was exposed or occluded for 14 min, followed by reperfusion for 1, 2, 3, or 7 days. The animals were sacrificed with pentobarbital and their L4–6 segments of spinal cord were collected for BSCB disruption evaluation, western blot, gelatin zymography, and immunofluorescence staining. In each group, five rats at each time point were used for hindlimb locomotor functional assessment, spinal cord edema measurement, western blot, and gelatin zymography; five rats at each time point were used for evaluation of Evans blue (EB) extravasation; and the other five rats were used for immunofluorescence staining.

### Surgical procedures

The spinal cord I/R model in rats was conducted according to the previously reported method [37]. Briefly, the rats were anesthetized with intraperitoneal administration of 4% sodium pentobarbital (50 mg/kg). The rectal temperature was monitored and kept at 37 ± 0.5 °C with a heated operating table. Catheters were placed into the tail artery and the left carotid artery for detecting the distal and proximal arterial blood pressure, respectively. The descending aorta was exposed with a left lateral thoracotomy. After systemic heparinization (200 IU/kg), under direct visualization, descending aorta was occluded with a cross-clamp just distal to the left subclavian artery. A reduction of the distal blood pressure to less than 10 mmHg was confirmed as ischemia. The cross-clamp was removed after a 14-min ischemia, followed by reperfusion. All rats were allowed to recover in a 37 °C incubator for 4 h and were subsequently put in separate cages with free access to food and water. Manual bladder expression was performed twice daily until recovery of autonomic function.

### Neurological assessment

The ability of rats to use their hindlimbs was investigated by an assessor blinded to the experimental protocol with Basso, Beattie, and Bresnahan (BBB) Locomotor Scale [38] over 4 min on post-reperfusion 1, 2, 3, and 7 days. The scoring criteria included joint movement, paw placement, and coordination. The rats with normal motor function obtained a BBB score of 21points, while the rats losing motor function completely were scored 0 point.

### BSCB disruption evaluation

The evaluation of BSCB integrity was performed with quantitative and qualitative measurements of EB extravasation and spinal cord edema. The content and fluorescence of EB were examined. Briefly, 60 min after the injection of EB dye (20 g/L; Sigma) at a dose of 10 ml/kg via the tail vein, the animals were anesthetized and then transcardially perfused with 0.9% saline containing 10 IU/ml heparin, until colorless perfusion fluid was obtained from the right atrium, to remove all EB dye that had not leaked into the interstitium. The L4–6 spinal cord tissue was weighed, then soaked with methanamide for 24 h (60 °C) and centrifuged. The fluorescent absorption of the supernatant was detected at 632 nm with a microplate reader (BioTek, Winooski, USA) and presented as EB amount per tissue weight (μg/g) with standardized curve. Furthermore, the spinal cord tissue was fixed with 4% paraformaldehyde. The transverse sections (10 μm) were visualized under a BX-61 (Olympus, JP) fluorescence microscope. EB dye was visualized as red under the green fluorescence excitation mode. The integrated optical density (IOD) of the positive fluorescence labeling in the lumbar spinal cord tissue was analyzed to quantify the EB extravasation (Image Pro Plus 6.0). The percentage of water content in the spinal cord tissue was calculated with a wet-dry method: (wet weight − dry weight)/wet weight × 100.

### Western blot analysis

The spinal cord tissue samples were extracted in RIPA lysis buffer, followed by centrifugation (12,000×*g*, 30 min, 4 °C). Protein concentration was measured with the BCA assay. Equal amounts of proteins (60 μg) were separated by 10% sodium dodecyl sulfate polyacrylamide gel electrophoresis (SDS-PAGE) and transferred onto a polyvinylidene fluoride (PVDF) membrane (Millipore, Billerica, MA, USA). The blots were blocked with 5% skim milk in Tris-buffered saline Tween (TBST) for 1 h at room temperature, and then incubated overnight at 4 °C with the following primary antibodies: rabbit polyclonal anti-cannabinoid receptor II (1:500, Abcam 3561), rabbit polyclonal anti-ZO-1 antibody (1:500, Invitrogen 40-2200), rabbit polyclonal anti-TLR4 (1:500, Abcam 13556), rabbit polyclonal anti-MMP9 (1:500, Abcam 38898), rabbit polyclonal anti-MyD88 (1:500, Abcam 2064), rabbit polyclonal anti-NF-κB p-p65 (1:1000, Abcam 86299), rabbit polyclonal anti- NF-κB p65 (1:1000, Abcam 16502), or rabbit monoclonal anti-GAPDH (1:1000, Abcam 8245). After washing, the membranes were incubated with horseradish peroxidase-conjugated secondary antibodies (Bioss, Beijing, China) for 2 h at room temperature. The bands were visualized by enhanced chemiluminescence (ECL Advance Kit; Bio-Rad). Protein expression was quantified by densitometric analysis (Image J). GAPDH was used as an internal loading control. The relative protein levels were normalized by the ratio of target protein to GAPDH.

### Gelatin zymography

The MMP9 activity was determined with gelatin zymography assay. Equal amounts of proteins (60 μg) were separated by 10% SDS-PAGE containing 0.1% gelatin (Invitrogen). The gel was washed in renaturing buffer (2.5% Triton X-100) (Invitrogen) for 1 h at room temperature. Then it was incubated overnight at 37 °C in development buffer (Invitrogen). Finally, the gel was stained with 0.25% R-250 Coomassie blue for 2 h and destained in 2: 1 methanol: acetic acid solution for 2 h at room temperature. The brightness of the clear bands, where the gelatin was degraded by MMP9, was subjected to densitometric analysis (Image J).

### Double immunofluorescence staining

The perivascular localization of CB2R, TLR4, MMP9, and reactive astrocytes were identified by double immunofluorescence labeling of CB2R, TLR4, MMP9, or reactive astrocytes marker glial fibrillary acidic protein (GFAP) with capillary endothelial cells marker CD31. Mouse monoclonal anti-CD31 [P2B1] (1:100, Abcam 24590) together with rabbit polyclonal anti-cannabinoid receptor II (1:500, Abcam 3561), rabbit polyclonal anti-TLR4 (1:100, Abcam 13556), rabbit polyclonal anti-MMP9 (1:100, Abcam 38898), or rabbit polyclonal anti-GFAP antibody (1:800, Abcam 7260) were used as primary antibodies to incubate the sections overnight at 4 °C, followed with corresponding secondary antibodies: Alexa 594-conjugated donkey anti-mouse IgG (1:200, Abcam 150108) and Alexa 488-conjugated donkey anti-rabbit IgG (1:200, Abcam 150073), each for 2 h at room temperature. The sections were imaged and analyzed by Leica TCS SP2 (Leica Microsystems, Buffalo Grove, IL, USA) laser scanning spectral confocal microscope. The average IOD of the positive fluorescence labeling in the gray matter of six transverse sections with ×40 magnification, at 140 μm intervals from 500 μm rostral and caudal to the center of the L4-6 spinal cord in each rat (*n* = 5 in each group), was analyzed to quantify the target protein expression (Image Pro Plus 6.0). Quantification of the perivascular protein expression is represented as the average IOD ratio of perivascular target protein to corresponding CD31 in six transverse sections at ×100 magnification in each rat (*n* = 5 in each group). Three blood vessels in the gray matter of each section were randomly selected by an assessor blinded to the experimental protocol.

For double staining of astrocytic marker with CB2R, TLR4, and MMP9, mouse monoclonal anti-GFAP antibody (1:800, Abcam 10062) together with rabbit polyclonal anti-cannabinoid receptor II (1:500, Abcam 3561), rabbit polyclonal anti-TLR4 (1:100, Abcam 13556), or rabbit polyclonal anti-MMP9 (1:100, Abcam 38898) were used as primary antibodies to incubate overnight at 4 °C, followed with corresponding secondary antibodies as mentioned above. The colocalization of target proteins with reactive astrocytes was quantified by the average IOD of the colocalization fluorescence labeling in the gray matter of six transverse sections with ×100 magnification in each rat (*n* = 5 in each group) (Image Pro Plus 6.0).

### Statistical analysis

The values were presented as means ± SEM and statistically analyzed with the SPSS 20.0 software. The data collected over time among the groups were compared by repeated measures ANOVA with time as within subject factor, group as between subject factor, followed by Bonferroni post-hoc tests. The other data were compared with Student’s *t* test or one-way ANOVA followed by Newman-Keuls post hoc analysis. Values of *P* < 0.05 were considered statistically significant.

## Results

### JWH-133 pretreatment attenuated hindlimb motor functional deficit and BSCB disruption after I/R injury, reversed by CB2R blocking

**Fig. 1:**
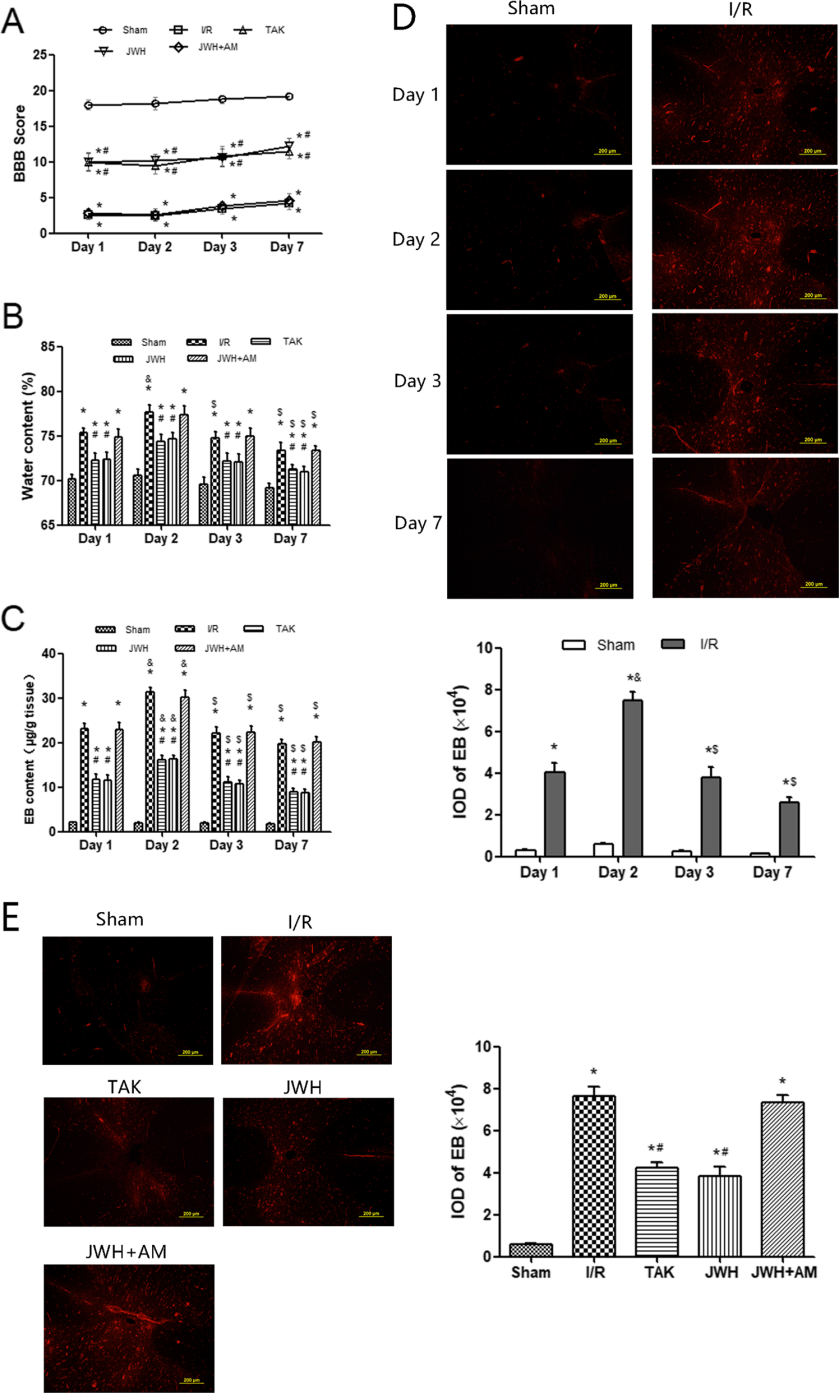
Effects of JWH-133 pretreatment on hindlimb motor function and BSCB permeability after I/R injury. **a** Neurological scores investigated by BBB Locomotor Scale at each time point during the 7 days observation after injury in each group. **b** Percentage of water content in the spinal cord tissue. **c** Amount of Evans blue (EB) in the spinal cord tissue. **d** Red fluorescence of EB extravasation in the spinal cord in the sham and I/R groups along the entire time course from 1 to 7 days after injury. Quantification of EB is represented as the average integrated optical density (IOD). Almost no red fluorescence displayed in the spinal cord of the sham group. I/R injury increased EB extravasation (red) at each observed time point, especially in the gray matter, with the maximal level at day 2 after injury. **e** Red fluorescence of EB extravasation in the spinal cord in each group at day 2 after injury. Quantification of EB is represented as the average IOD. In comparison to I/R group, EB extravasation was obviously lower in JWH or TAK group, especially in the gray matter. AM630 pretreatment diminished the effects of JWH-133. All results are represented as mean ± SEM (*n* = 5 per group at each time point). *P < 0.05 vs. sham group; #*P* < 0.05 vs. I/R group; & *P* < 0.05 vs. day 1; $ *P* < 0.05 vs. day 2. Scale bars are 200 μm

No motor functional deficit was observed in sham group rats, while the rats that underwent I/R protocol displayed various degrees of motor deficit during the 7 days of evaluation using the BBB scoring system (*P* < 0.05 vs. sham group) (Fig. 1a). JWH-133 pretreatment improved the BBB scores in comparison to I/R group at each time point (*P* < 0.05). The AM630 reversed the recovery of neurological function in JWH-133 group rats. The scores in JWH + AM and I/R groups showed no statistical difference (*P* > 0.05). Similar to the effects of JWH-133 preconditioning, pretreatment with TLR4 inhibitor TAK-242 was observed to result in statistically higher scores when contrasted to IR group at each time point (*P* < 0.05). The BBB scores over time course within groups showed no statistical difference (*P* > 0.05).

I/R injury caused significant increase in water content contrasted with that of sham group during 7 days after reperfusion (*P* < 0.05) (Fig. 1b), which was closely related to spinal cord edema following BSCB breakdown. JWH-133 preconditioning relieved the spinal cord edema after I/R injury at each time point (*P* < 0.05 vs. I/R group), which was antagonized by AM630 pretreatment (*P* > 0.05 vs. I/R group). TAK-242 pretreatment showed comparable effects on reducing spinal cord water content as JWH-133 pretreatment (*P* > 0.05). Furthermore, our results revealed that the most severe spinal cord edema occurred at day 2 after injury. The water content of I/R group at day 2 was significantly higher than that at other time points (*P* < 0.05). Similarly, the water content of TAK, JWH, or JWH + AM group at day 2 was significantly higher than that at day 7 (*P* < 0.05), and there was an increasing trend compared with that at day 1 or day 3, although the significance was not achieved (*P* > 0.05).

To evaluate the disruption of the BSCB, EB extravasation content in spinal cord tissue was assessed quantitatively and qualitatively. Consistent with the degree of spinal cord edema, the quantitative analysis of EB content at each time point displayed that I/R injury significantly increased the EB content in spinal cord tissue contrasting to sham group (*P* < 0.05), and the maximal level occurred at day 2 after injury (*P* < 0.05 vs. other time points) (Fig. 1c), which was also shown with EB extravasation fluorescence and quantification of red fluorescence with IOD (Fig. 1d). Almost no red fluorescence displayed in the spinal cord of the sham group. I/R injury increased EB extravasation (red) at each observed time point, especially in the gray matter, with the maximal level at day 2 after injury. JWH-133 or TAK-242 pretreatment displayed obvious attenuation of BSCB leakage (*P* < 0.05 vs. I/R group). CB2R antagonist AM630 reversed the beneficial effect of JWH-133 pretreatment on BSCB (*P* > 0.05 vs. I/R group). The EB extravasation visualized with fluorescent microscope (red fluorescence) and quantified with IOD displayed similar trend at day 2 after injury (Fig. 1e), the time point with the maximal spinal water and EB content.

### JWH-133 pretreatment prevented downregulation of ZO-1 and upregulation of TLR4/MMP9 after I/R injury

**Fig. 2:**
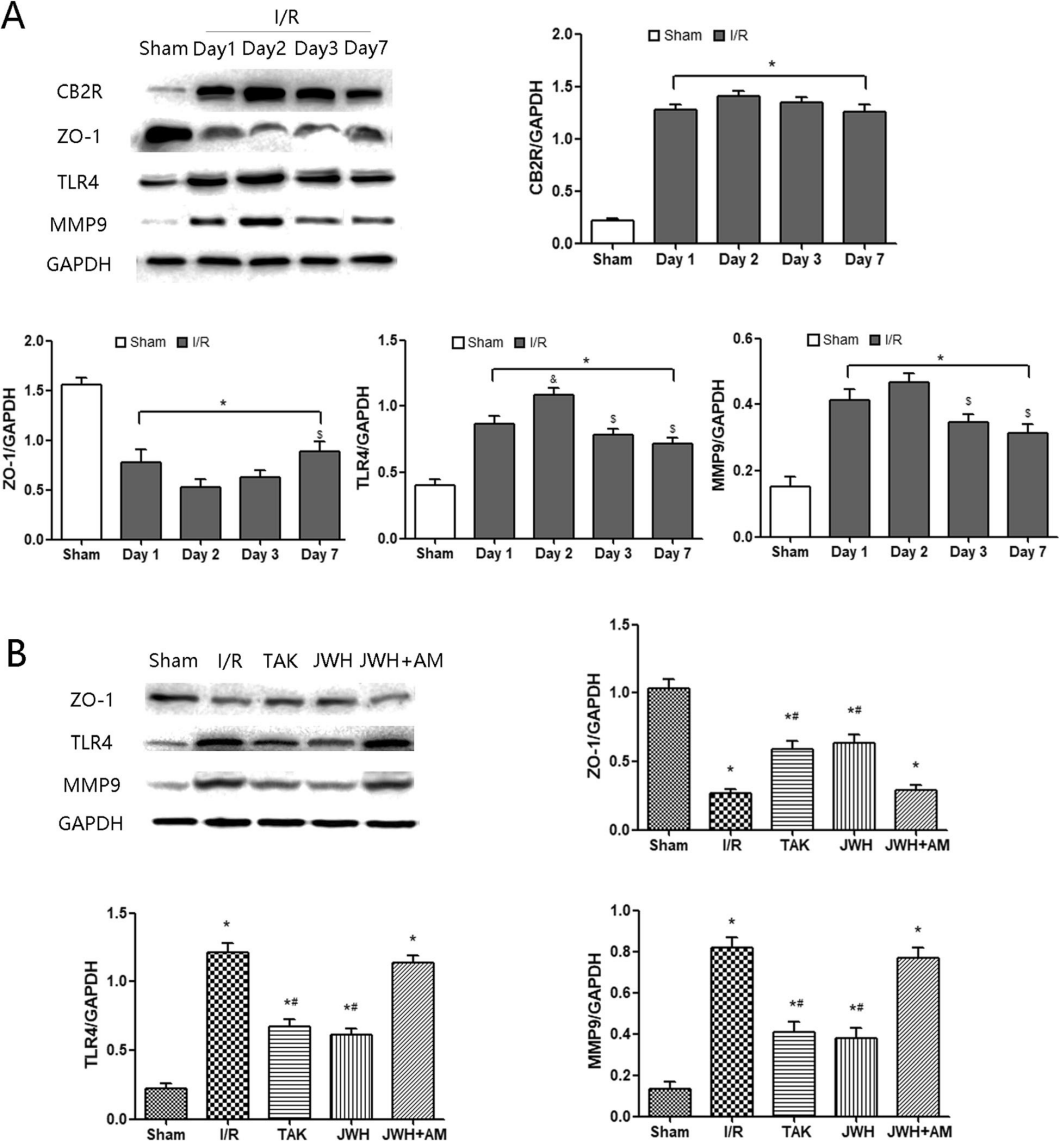
Effects of JWH-133 pretreatment on the protein expression of ZO-1, TLR4, and MMP9 after I/R injury in the spinal cord tissue. **a** Protein expression of CB2R, ZO-1, TLR4, and MMP9 in the sham and I/R groups along the entire time course from 1 to 7 days after injury. I/R injury resulted in downregulation of ZO-1 and upregulation of TLR4/MMP9 during 7 days after injury, and this effect was most obvious at day 2 after injury. The expression of CB2R following I/R injury showed a sustained high level from day 1 to day 7 after injury. b Protein expression of ZO-1, TLR4, and MMP9 in each group at day 2 after injury. JWH-133 or TAK-242 treatment increased the ZO-1 protein level and reduced the level of TLR4/MMP9 comparing with I/R group. All results are represented as mean ± SEM (*n* = 5 per group at each time point). *P < 0.05 vs. sham group; #*P* < 0.05 vs; & *P* < 0.05 vs. day 1; $ *P* < 0.05 vs. day 2. I/R group

The protein levels of ZO-1, TLR4, MMP9, and CB2R in lumbar spinal cord tissue were investigated by western blot. I/R injury resulted in downregulation of ZO-1 and upregulation of TLR4/MMP9 during 7 days after injury (*P* < 0.05 vs. sham group), and this effect was most obvious at day 2 after injury (ZO-1: *P* < 0.05 vs. day 7; TLR4: *P* < 0.05 vs. other time points; MMP9: *P* < 0.05 vs. day 3 or day 7) (Fig. 2a). The expression of CB2R following I/R injury showed a sustained high level from day 1 to day 7 after injury (*P* < 0.05 vs. sham group, *P* > 0.05 between time points). We determined the effects of JWH-133 pretreatment and TLR4 pathway on the expression of ZO-1, TLR4, and MMP9 at day 2 after injury. JWH-133 or TAK-242 treatment increased the ZO-1 protein level and reduced the level of TLR4/MMP9 comparing with I/R group (*P* < 0.05) (Fig. 2b), suggesting JWH-133 or TAK-242 pretreatment prevented the downregulation of ZO-1 and upregulation of TLR4/MMP9 following I/R injury.

### JWH-133 pretreatment prevented upregulation of MyD88/NF-κB and activation of MMP9 after I/R injury

**Fig. 3:**
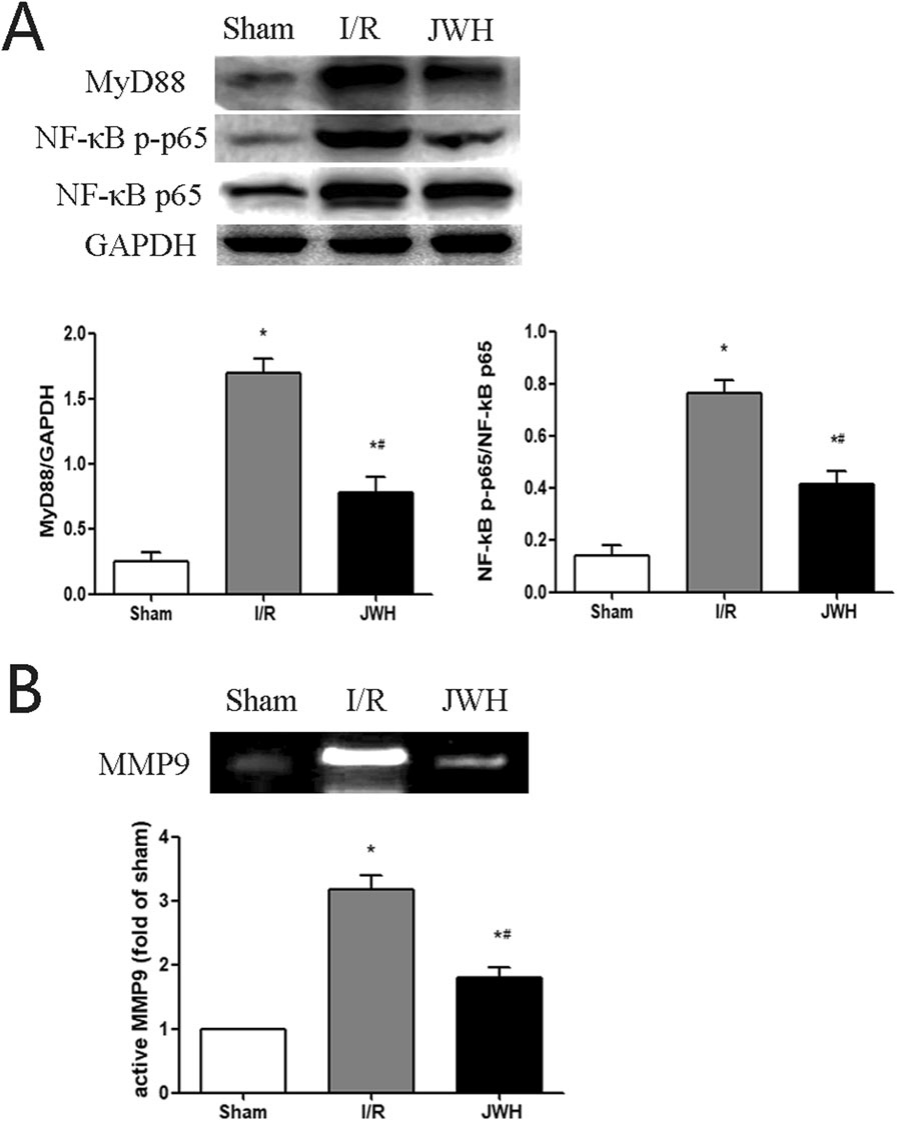
Effects of JWH-133 pretreatment on the protein expression of MyD88/NF-κB and MMP9 activity after I/R injury in the spinal cord tissue. **a** Protein expression of MyD88, NF-κB p-p65, and NF-κB p65 at day 2 after injury. JWH-133 treatment reduced the expression of MyD88 and NF-κB p-p65 comparing with I/R group. **b** MMP9 activity at day 2 after injury. The MMP9 activity of JWH group was obviously less than that of I/R group. All results are represented as mean ± SEM (*n* = 5). *P < 0.05 vs. sham group; #*P* < 0.05 vs. I/R group

Via MyD88-dependent pathway, TLR4 provokes downstream NF-κB signaling, which was involved in the activation of MMP9 [21, 26]. We further detected the expression of MyD88, NF-κB p-p65, and NF-κB p65 in spinal cord tissue by western blot, as well as MMP9 activity by gel zimography at day 2 after injury. I/R injury induced upregulation of MyD88 and NF-κB p-p65 significantly (*P* < 0.05 vs. sham group). JWH-133 treatment reduced the expression of MyD88 and NF-κB p-p65 comparing with I/R group (*P* < 0.05) (Fig. 3a). Similarly, I/R injury-enhanced MMP9 activity (*P* < 0.05 vs. sham group), which could be prevented by JWH-133 pretreatment (*P* < 0.05 vs. I/R group) (Fig. 3b).

### JWH-133 pretreatment prevented upregulation of TLR4/MMP9 perivascular expression after I/R injury

**Fig. 4:**
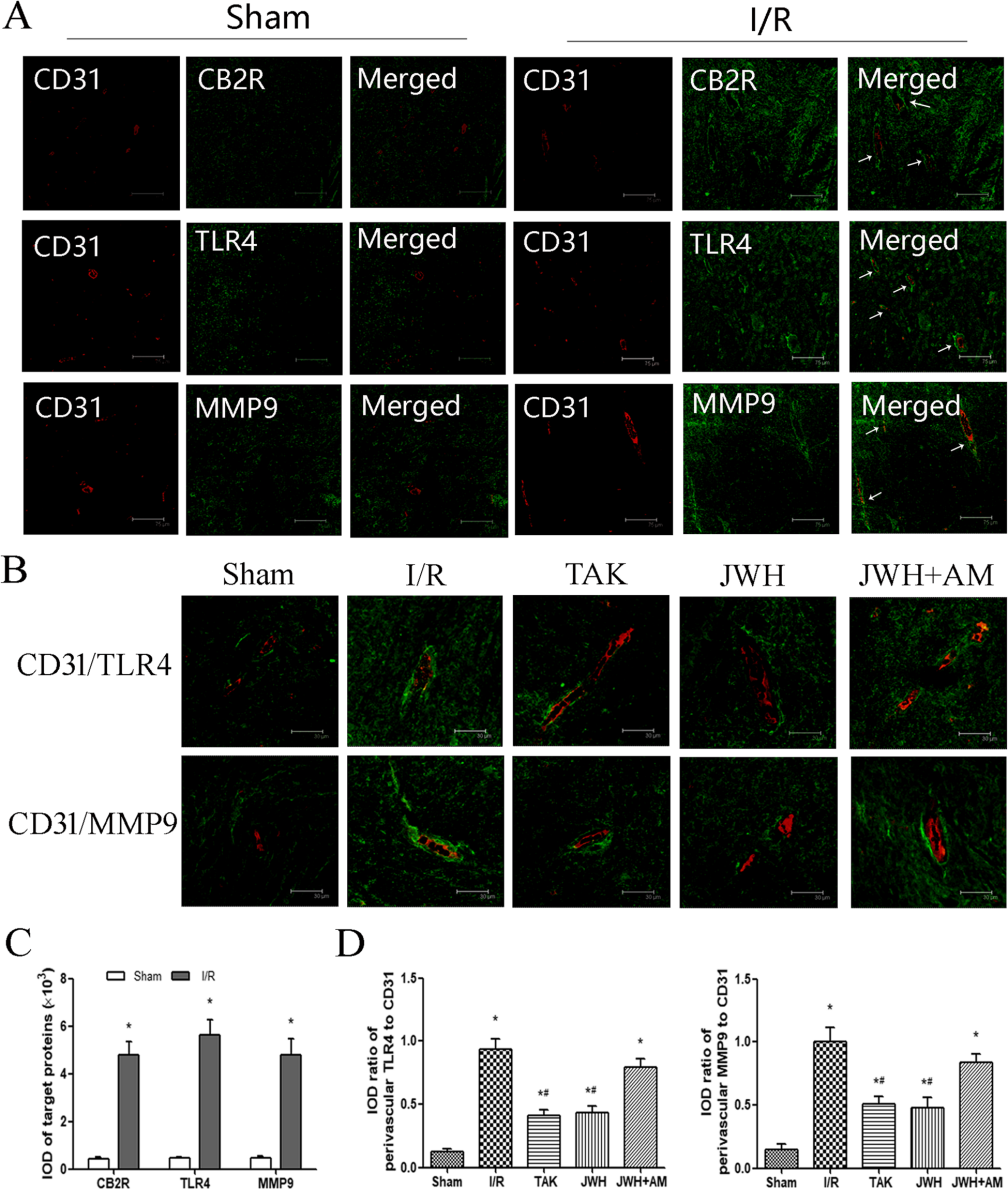
Effects of JWH-133 pretreatment on the perivascular expression of TLR4 and MMP9 after I/R injury in the gray matter of spinal cord. **a** Representative immunofluorescence images of CB2R (green), TLR4 (green), and MMP9 (green) colocalized with capillary endothelial cells (CD31; red) in the Sham and I/R groups at day 2 after injury. Arrows show the perivascular expression of CB2R, TLR4, and MMP9. Scale bars are 75 μm. **b** Higher magnification images of TLR4 (green) and MMP9 (green) colocalized with capillary endothelial cells (CD31; red) in each group at day 2 after injury. Scale bars are 30 μm. **c** Quantification of CB2R, TLR4, and MMP9 is represented as the average IOD. CB2R, TLR4, and MMP9 positive fluorescence labeling significantly increased in I/R group contrasting to that in sham group. **d** Quantification of TLR4/MMP9 perivascular expression is represented as the average IOD ratio of perivascular TLR4/MMP9 to corresponding CD31. I/R injury increased TLR4/MMP9 perivascular expression compared to sham group. The positive fluorescence labeling of TLR4/MMP9 along microvessels were obviously lower in JWH or TAK group compared to I/R group. All results are represented as mean ± SEM (*n* = 5). *P < 0.05 vs. sham group; #*P* < 0.05 vs. I/R group

To confirm our hypothesis, we detected the perivascular localization of CB2R, TLR4, and MMP9 in the L4-6 spinal cord tissue with immunofluorescence at day 2 after injury. The immunofluorescence labeling of CB2R (green), TLR4 (green), MMP9 (green), and capillary endothelial cells (CD31; red) in the gray matter was displayed in Fig. 4a. Similar to the protein levels detected by western blot, CB2R, TLR4, and MMP9 positive fluorescence labeling significantly increased in I/R group contrasting to that in sham group, which was confirmed by the quantification of the target proteins IOD (*P* < 0.05) (Fig. 4c). Some of the positive labeling localized around the microvessels (Fig. 4a, arrows). The higher magnification images of double immunofluorescence with TLR4 (green), MMP9 (green), and CD31 (red) were shown as Fig. 4b. The perivascular expression level of TLR4/MMP9 was quantified with the average IOD ratio of perivascular target protein to corresponding CD31 (Fig. 4d). I/R injury induced the upregulation of TLR4/MMP9 perivascular expression (*P* < 0.05 vs. sham group). JWH-133 pretreatment obviously reduced the expression level of TLR4/MMP9 along the microvessels compared to I/R group (*P* < 0.05), which was reserved by AM630 treatment (*P* > 0.05 vs. I/R group). TAK-242 pretreatment revealed the comparable effects with the JWH-133 preconditioning (*P* > 0.05).

### JWH-133 pretreatment inhibited perivascular activation of astrocytes after I/R injury

**Fig. 5:**
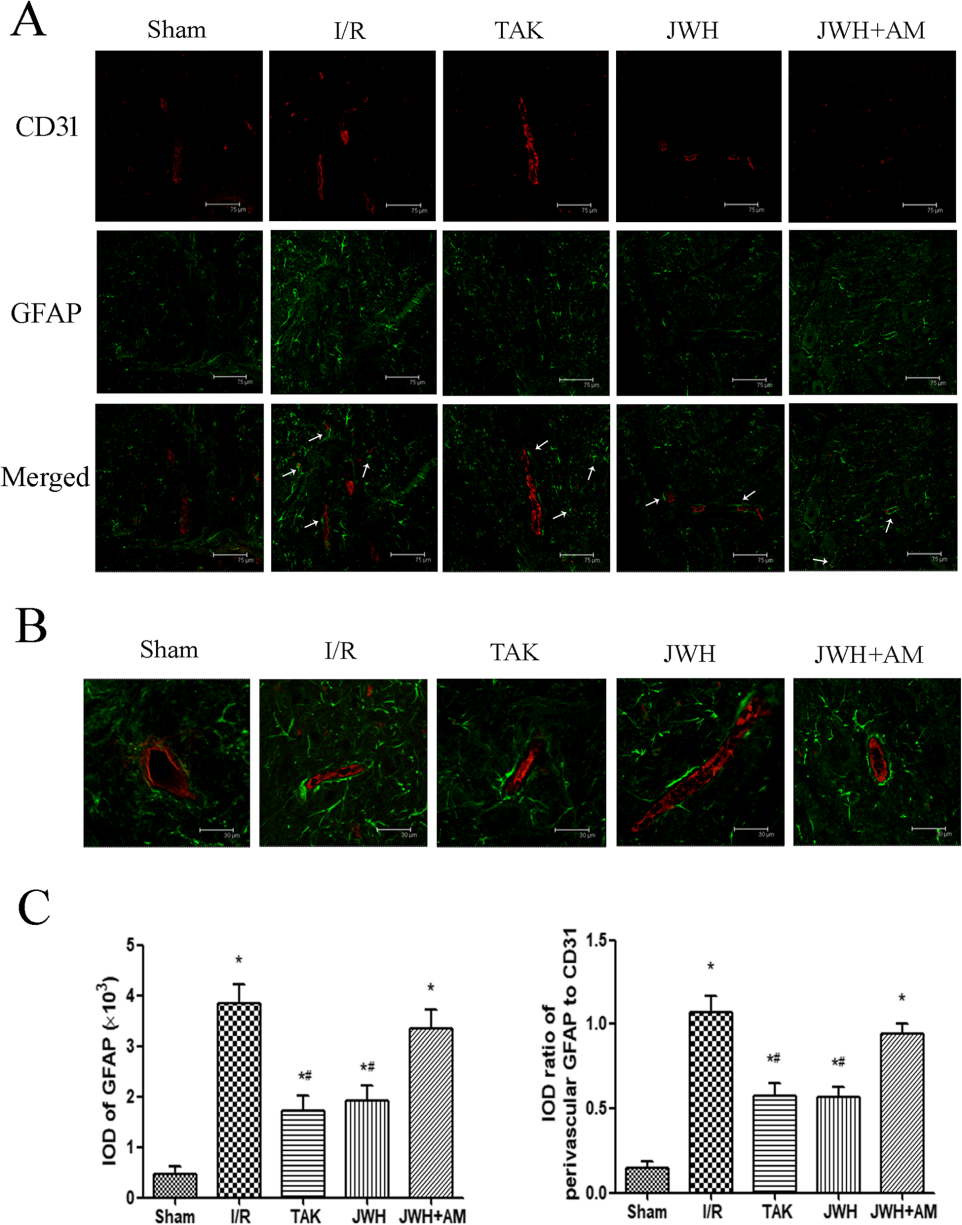
Effects of JWH-133 pretreatment on the perivascular activation of astrocytes after I/R injury in the gray matter of spinal cord. **a** Representative immunofluorescence images of reactive astrocytes (GFAP; green) colocalized with capillary endothelial cells (CD31; red) at day 2 after injury. Arrows show the perivascular expression of GFAP. Scale bars are 75 μm. **b** Higher magnification images of reactive astrocytes (GFAP; green) colocalized with capillary endothelial cells (CD31; red) at day 2 after injury. Scale bars are 30 μm. **c** Quantification of GFAP or its perivascular expression is represented as the average IOD or IOD ratio of perivascular GFAP to corresponding CD31. The positive fluorescence labeling of GFAP in the spinal cord and that along microvessels both increased after I/R injury, which could be downregulated by JWH-133 or TAK-242 pretreatment. All results are represented as mean ± SEM (*n* = 5). *P < 0.05 vs. sham group; #*P* < 0.05 vs. I/R group

Increased GFAP expression is the best known hallmark of reactive astrocytes. To evaluate the perivascular activation of astrocytes, we double labeled the GFAP with capillary endothelial cell marker CD31 in the L4-6 spinal cord tissue at day 2 after injury. The double immunofluorescence with GFAP (green) and CD31 (red) in the gray matter was shown in Fig. 5a. The arrows point to the GFAP around the microvessels. The representative higher magnification images were shown as Fig. 5b. Quantification with the IOD of GFAP confirmed that the astrocytes in the spinal cord tissue were activated in I/R injury (*P* < 0.05 vs. sham group), which could be suppressed by JWH-133 or TAK-242 treatment (*P* < 0.05 vs. I/R group). AM630 pretreatment diminished the effects of JWH-133 on astrocytes activation (*P* > 0.05 vs. I/R group) (Fig. 5c). The expression of reactive astrocytes around the microvessels quantified with the average IOD ratio of perivascular GFAP to corresponding CD31 showed similar trend (Fig. 5c), indicating JWH-133 pretreatment prevented upregulation of reactive astrocytes perivascular expression following I/R injury.

### JWH-133 pretreatment downregulated the astrocytic TLR4/MMP9 after I/R injury

**Fig. 6:**
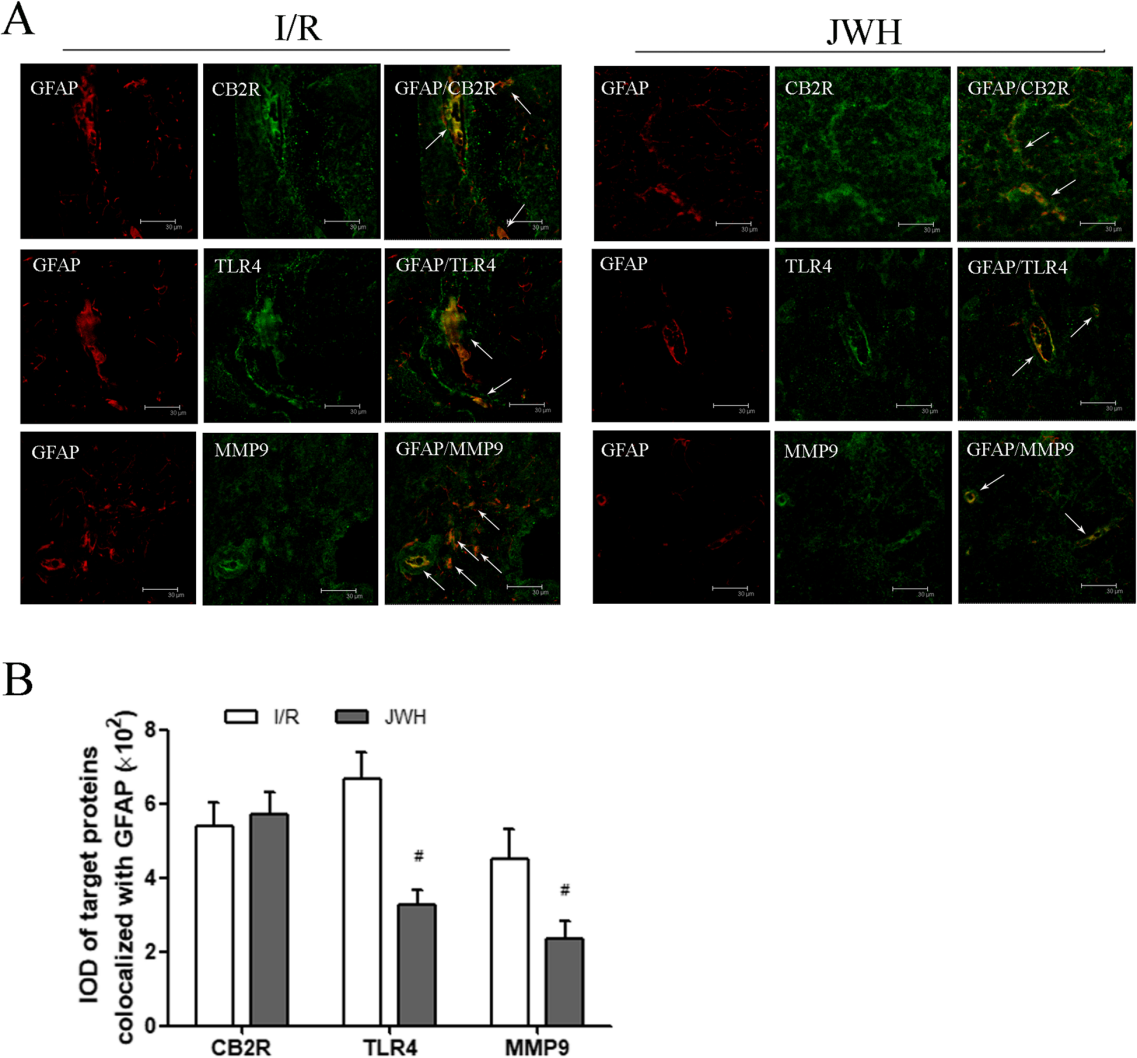
Effects of JWH-133 pretreatment on the colocalization of CB2R, TLR4, and MMP9 with reactive astrocytes after I/R injury in the gray matter of spinal cord. **a** Representative immunofluorescence images of CB2R (green), TLR4 (green), and MMP9 (green) colocalized with reactive astrocytes (GFAP; red) at day 2 after injury. The arrows show the colocalization fluorescence labeling (yellow). Scale bars are 30 μm. **b** Quantification of the colocalization expression for target proteins with reactive astrocytes is represented as the average IOD of the colocalization fluorescence labeling. The astrocytic TLR4/MMP9 in JWH group was significantly less than that in I/R group. All results are represented as mean ± SEM (*n* = 5). #*P* < 0.05 vs. I/R group

We colocalized CB2R, TLR4, and MMP9 with reactive astrocytes by double staining of target proteins with GFAP in the L4-6 spinal cord tissue at day 2 after injury. The immunofluorescence labeling of CB2R (green), TLR4 (green), MMP9 (green), and reactive astrocytes (GFAP; red) in the gray matter was showed in Fig. 6a. The arrows point to the colocalization of target proteins with reactive astrocytes (yellow), which was quantified by the average IOD of the colocalization fluorescence labeling (Fig. 6b). JWH-133 pretreatment significantly reduced the expression of astrocytic TLR4/MMP9 compared to I/R group (*P* < 0.05), whereas had no obvious effect on CB2R colocalized with astrocytes (*P* > 0.05 vs. I/R group).

## Discussion

The present study showed several novel outcomes: (1) CB2R agonist JWH-133 attenuated BSCB disruption, spinal cord edema, and improved neurological function after spinal cord I/R injury. (2) JWH-133 prevented downregulation of ZO-1 and upregulation of perivascular reactive astrocytes following I/R injury. (3) The beneficial effects of JWH-133, at least in part, involved the TLR4/MMP9 pathway.

Currently, hypothermia and cerebrospinal fluid drainage are clinically effective for prevention and treatment of spinal cord ischemic injury in clinical thoracoabdominal aneurysm repair. The medical options available for spinal cord protection are extremely limited. Modulation of the receptor-mediated endocannabinoid system can produce neuroprotective effects during spinal cord injury [11, 39]. Our study revealed that administration of CB2R agonist JWH-133 improved motor function and preserved BSCB integrity over the 7-day period of observation in a spinal cord I/R rat model, which could be blocked by CB2R antagonist AM630, corresponding with the previous study [40, 41]. In the current study, we utilized the rat spinal cord I/R injury model with 14-min occlusion of descending aorta [37], which is much closer to clinical thoracoabdominal aneurysm repair than I/R rat model conducted with transient occlusion of abdominal aorta [22] and the murine spinal cord contusion model [16].

One of the mechanisms via which CB2R agonist treatment may contribute to preservation of BSCB integrity is modulation of inflammatory responses following spinal cord injury [16]. TLR4 and MMP9 are the important mediators in inflammation-induced BSCB breakdown [5, 15, 28]. TLR4 elevation induces astrocytes polarization [24]. The reactive astrocytes around microvessels activate endothelial MMP9 [25] or release inflammatory mediators such as cytokines IL-1β, IL-6, and TNFα to regulate blood-brain barrier permeability in inflammation [42]. The MMP-9 activation is responsible for the degradation of tight junction proteins and the opening of tight junctions [34], which leads to the increase of endothelial permeability [3]. In the current investigation, we were able to show that exogenous activation of CB2R prevented upregulation of TLR4/MMP9 expression after I/R injury, and proportionally prevented perivascular activation of astrocytes and downregulation of ZO-1, followed by improvement of BSCB permeability and neurological function, which were also found in rats with TAK-242 administration. Therefore, it can be assumed that exogenous activation of CB2R alleviates spinal cord I/R injury by regulating TLR4/MMP9 expression.

The myeloid differentiation factor 88 (MyD88) is the central adaptor protein of TLR4 signal pathway, and facilitates the transduction of downstream signaling molecules, such as NF-κB, to regulate the expression of proinflammatory cytokines and chemokines [43–45]. In addition, it has been suggested that the activation of MMP9 is modulated by NF-κB phosphorylation [28, 46]. To confirm that the exogenous activation of CB2R in the context of I/R injury directly inhibit TLR4 signaling to modulate MMP9 activity, we further analyzed the expression levels of MyD88 and NF-κB phosphorylationin response to the JWH-133 treatment, as well as MMP9 activity by gel zimography. We found JWH-133 pretreatment prevented the activation of MyD88/NF-κB and MMP9 following I/R injury, suggesting that the exogenous activation of CB2R inhibited TLR4/MyD88/NF-κB signaling to reduce MMP9 activity. Previous studies have reported that intrathecal injection of specific siRNAs targeting MMP9 to reduce its activity and expression could improve BSCB integrity in spinal cord I/R injury [8, 47, 48].

However, matrix metalloproteinases (MMPs) play a dual role by acutely disrupting the tight junction proteins in the blood-brain barrier and chronically promoting angiogenesis [49]. Recent study showed MMPs promote the activation and migration of astrocytes to form protected zone and reduce the cerebral infarct volume after ischemic stroke [50]. It has been reported that in central nervous system injuries, there are at least two types of reactive astrocytes A1 and A2, one type being helpful and the other harmful [51]. In the present study, we did not identify the reactive astrocytes as A1 or A2, but found that the reactive astrocytes are involved in the destruction of BSCB.

This study confirmed perivascular localization of CB2R, TLR4, and MMP9 by double immunofluorescence labeled with capillary endothelial cells marker CD31. Moreover, the expression of TLR4/MMP9 and reactive astrocytes along spinal microvessels after I/R injury were all reduced by CB2R activation. These findings revealed the close apposition of CB2R, TLR4, MMP9, reactive astrocytes, and spinal microvessels, which providing geographic evidence for CB2R activation modulating perivascular expression of TLR4/MMP9 and reactive astrocytes to preserve tight junctions and endothelial integrity. Moreover, the findings also indicated that reactive astrocytes may be the key player in the CB2R/TLR4/MMP9 axis. So, we further detected the colocalization of CB2R, TLR4, and MMP9 with reactive astrocytes. As expected, CB2R, TLR4, and MMP9 colocalized with reactive astrocytes after I/R injury, and JWH-133 treatment significantly decreased the astrocytic TLR4/MMP9 expression compared to I/R group, which may provide evidence for JWH-133 working on reactive astrocytes to regulate TLR4/MMP9 expression. Previous studies have shown that CB2R is located in glial cells, and its expression increases in response to neuroinflammation [11, 14, 52, 53]. Both glial TLR4 and MMP9 pathways are involved in the inflammation following I/R injury [8, 15]. So, we may also look into microglia or perivescular fibroblasts to prove the specificity of cell type that JWH-133 works on in the further research.

CB2R activation showed the beneficial effects on spinal cord I/R injury throughout the entire observation period in this study, which was consistent with the high expression of CB2R after injury. We evaluated the expression of CB2R in the spinal cord tissue, which showed a sustained increase from day 1 to day 7 after injury. Previous studies also suggested that the ischemic injury induced a delayed increase of CB2R expression at 24 h after injury [13, 14], and the increased expression of CB2R could last for 28 days after spinal cord injury [11]. The endocannabinoids act through CB1 and CB2 cannabinoid receptors to display a protective response involved in spontaneous recovery up to 90 days after spinal cord injury [39]. Therefore, a longer observation time is essential to evaluate the effects of CB2R activation on the secondary injury and the spontaneous recovery after spinal cord I/R injury.

Considering that prevention is better than treatment, we tried pretreatment in the current study, expecting the protective effects before acute ischemic injury and secondary inflammatory injury. The findings, as expected, showed that pretreatment with CB2R agonist JWH-133 reduced spinal I/R injury, which is similar to those previous reports [22, 54]. However, post injury administration is also clinically relevant due to some unpredictable spinal cord injuries. Yu et al. indicated that 48 h post-stroke treatment with a CB2R agonist did not efficiently suppress brain damages, while preconditioning of the CB2R agonist significantly reduced the infarct volume [55]. A similar report has also showed that CB2R agonists, given either before or 10 min after the beginning of ischemia, reduced the microglial activation, the area of infarction, and neurological symptoms [17], suggesting that early treatment with CB2R agonists may attenuate ischemic cerebral injury. In our previous study, we observed the disrupted BSCB at 4 h after reperfusion [12], which may aggravate injury, resulting in paraplegia. Treatment with a selective CB2R agonist 1 h post spinal cord contusion injury also revealed beneficial effects [16]. Therefore, we predict that post injury administration of JWH-133 before BSCB disruption could alleviate BSCB leakage and neurological deficit following I/R injury, which needs further studies to confirm.

## Conclusions

In summary, the current study demonstrates that exogenous activation of CB2R attenuates neurological deficit and BSCB disruption after spinal cord I/R injury via inhibition of TLR4/MMP9 expression. These findings suggest that CB2R may represent a therapeutic target for spinal cord I/R injury.

## Data Availability

Data supporting the conclusions of this article are presented in the manuscript.

## Abbreviations

CB2R: Cannabinoid-2 receptor
TLR4: Toll-like receptors 4
MMP9: Matrix metalloproteinase 9
BSCB: Blood-spinal cord barrier
I/R: Ischemia reperfusion
BBB: Basso, Beattie, and Bresnahan
EB: Evans blue
IOD: Integrated optical density
GFAP: Glial fibrillary acidic protein
CD31: Platelet endothelial cell adhesion molecule-1
